# Ellagic Acid Prevented Dextran-Sodium-Sulfate-Induced Colitis, Liver, and Brain Injury through Gut Microbiome Changes

**DOI:** 10.3390/antiox12101886

**Published:** 2023-10-20

**Authors:** Dong-ha Kim, Ji-Su Kim, Jae-Hee Kwon, In-Sook Kwun, Moon-Chang Baek, Gi-Seok Kwon, Wiramon Rungratanawanich, Byoung-Joon Song, Do-Kyun Kim, Hyo-Jung Kwon, Young-Eun Cho

**Affiliations:** 1 Department of Molecular Medicine, School of Medicine, Cell & Matrix Research Institute, Kyungpook National University, Daegu 41566, Republic of Korea; a960112@knu.ac.kr (D.-h.K.); mcbaek@knu.ac.kr (M.-C.B.); 2 Department of Food and Nutrition, Andong National University, Andong 1375, Republic of Korea; 20235028@student.anu.ac.kr (J.-S.K.); 20235243@anu.ac.kr (J.-H.K.); iskwun@anu.ac.kr (I.-S.K.); 3 Department of Horticulture & Medicinal Plant, Andong National University, Andong 1375, Republic of Korea; gskwon@anu.ac.kr; 4 Section of Molecular Pharmacology and Toxicology, Laboratory of Membrane Biochemistry and Biophysics, National Institute on Alcohol Abuse and Alcoholism, National Institutes of Health, Bethesda, MD 20892, USA; wiramon.rungratanawanich@nih.gov (W.R.); bj.song@nih.gov (B.-J.S.); 5 Korea Zoonosis Research Institute, Jeonbuk National University, Iksan 54596, Republic of Korea; dkkim714@jbnu.ac.kr; 6 Department of Veterinary Pathology, College of Veterinary Medicine, Chungnam National University, Daejeon 34134, Republic of Korea

**Keywords:** inflammatory bowel disease, dextran sulfate sodium, ellagic acid, NF-κB/MAPK activation, anti-inflammation, antioxidant

## Abstract

Inflammatory bowel disease (IBD) affects millions of people worldwide and is considered a significant risk factor for colorectal cancer. Recent in vivo and in vitro studies reported that ellagic acid (EA) exhibits important antioxidant and anti-inflammatory properties. In this study, we investigated the preventive effects of EA against dextran sulfate sodium (DSS)-induced acute colitis, liver, and brain injury in mice through the gut–liver–brain axis. Acute colitis, liver, and brain injury were induced by treatment with 5% (*w*/*v*) DSS in the drinking water for 7 days. Freshly prepared EA (60 mg/kg/day) was orally administered, while control (CON) group mice were treated similarly by daily oral administrations with a vehicle (water). All the mice were euthanized 24 h after the final treatment with EA. The blood, liver, colon, and brain samples were collected for further histological and biochemical analyses. Co-treatment with a physiologically relevant dose (60 mg/kg/day) of EA for 7 days significantly reduced the DSS-induced gut barrier dysfunction; endotoxemia; and inflammatory gut, liver, and brain injury in mice by modulating gut microbiota composition and inhibiting the elevated oxidative and nitrative stress marker proteins. Our results further demonstrated that the preventive effect of EA on the DSS-induced IBD mouse model was mediated by blocking the NF-κB and mitogen-activated protein kinase (MAPK) pathway. Therefore, EA co-treatment significantly attenuated the pro-inflammatory and oxidative stress markers by suppressing the activation of NF-κB/MAPK pathways in gut, liver, and brain injury. These results suggest that EA, effective in attenuating IBD in a mouse model, deserves further consideration as a potential therapeutic for the treatment of inflammatory diseases.

## 1. Introduction

Inflammatory bowel disease (IBD), including Crohn’s disease (CD) and ulcerative colitis (UC), is a chronic, relapsing, inflammatory disease of the gastrointestinal (GI) tract [1]. Although the etiology of IBD has not been fully elucidated, recent reports indicate that environmental, genetic, and immunological conditions are being recognized as important risk factors [2]. The clinical features of IBD are presented by abdominal pain, diarrhea, bleeding, and bloody stool due to ulceration of the colon and rectum, being characterized by chronic infection with repeated cycles of recurrence and recovery [3]. Chronic inflammation has been reported to be associated with the pathogenesis of various human inflammatory diseases such as cancer, arthritis, asthma allergies, ulcerative colitis, and IBD [4,5,6].

Inflammatory responses are generally accelerated by pro-inflammatory cytokines and enzymes such as TNF, IL-1β, IL-6, COX-2, and iNOS, following the activation of NF-κB, a key pro-inflammatory transcription factor that is known to be activated under oxidative stress. NF-κB can also be stimulated through activated mitogen-activated protein kinases (MAPKs), including phosphorylation of p38, ERK1/2, and JNK [7]. Given the pivotal roles that pro-inflammatory mediators have in the etiologic factors associated with IBD, the method to reduce the levels of these inflammatory mediators is considered an effective therapeutic strategy for improving IBD [8]. Therefore, the treatment aims for IBD include rapid induction and steroid-free maintenance of clinical remission, as well as the effective control of chronic inflammation for mucosal healing [9]. In fact, some drugs used in IBD treatment, including sulfasalazine (mesalazine), aminosalicylate-based drug (5-ASA), and corticosteroids (prednisolone, methylprednisolone, and budesonide), are reported to inhibit inflammation response [10]. However, the use of these drugs has clinical limitations due to their side effects and high relapse rates [11]. Consequently, the current study focused on alternative or adjunct therapies that can reduce the shortcomings and side effects of the drugs currently being used to treat IBD.

**Figure 1:**
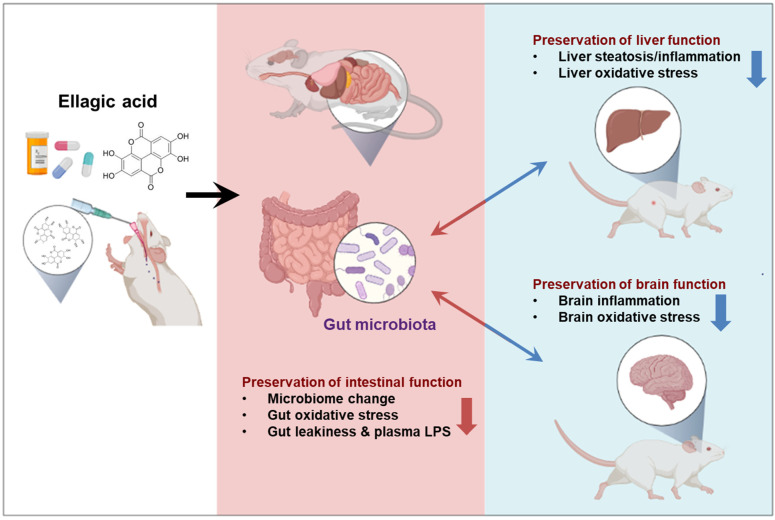
Schematic summary of the molecular mechanisms by which ellagic acid prevents DSS-induced microbial changes, gut oxidative stress/inflammation marker proteins, and intestinal hyper-permeability with elevated plasma LPS, thereby contributing to reduced liver and brain damage.

Numerous studies have reported the beneficial role of dietary polyphenols, secondary plant metabolites universally present in various fruits and vegetables, in managing several autoimmune diseases, including IBD [12,13]. Ellagic acid (EA), a polyphenolic compound from the family of ellagitannins (Ets), is present in many fruits (e.g., pomegranates, persimmons, raspberries, black raspberries, wild strawberries, peaches, plums), seeds (walnuts, almonds), and vegetables [14]. Over the past several years, many in vivo and in vitro studies have reported that EA exhibits important pharmacological properties, including antioxidant [15], anti-inflammatory [16], and anticancer [17] properties. Despite several studies about the beneficial effects of EA, only a limited number of studies have investigated the anti-inflammatory effects and protective mechanisms of EA on dextran sulfate sodium (DSS)-induced IBD model in mice. Therefore, in the present study, we investigated the pharmaceutical effects of EA on a few inflammatory markers and NF-κB/MAPK signaling pathways in DSS-induced gut, liver, and brain injury in mice through the gut–liver–brain axis [18,19,20] (Figure 1).

## 2. Materials and Methods

### 2.1. Materials

EA used in this study was purchased from Sigma Chemical (St. Louis, MO, USA). Other materials were used same information as recently described [19,21,22,23,24].

### 2.2. Animals

**Figure 2:**
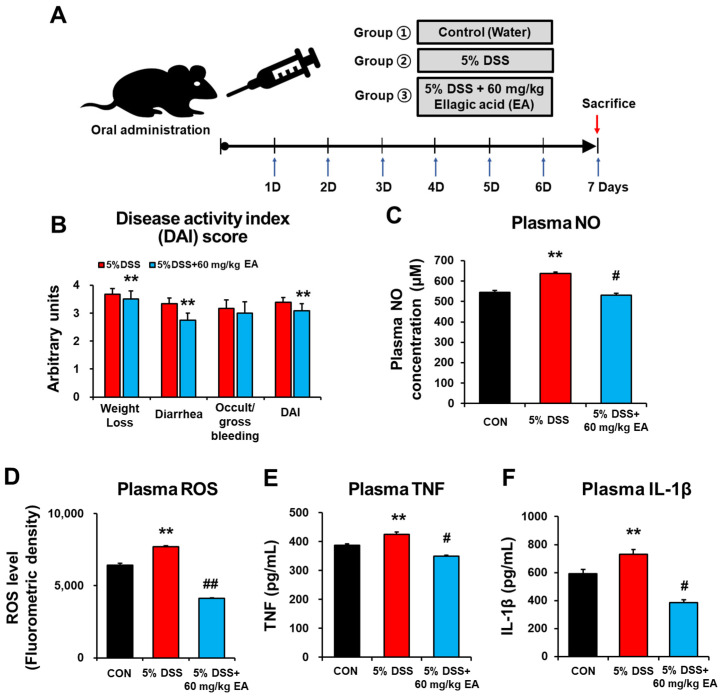
EA attenuated the progression of DSS-induced IBD mice by antioxidative and anti-inflammatory effects. (**A**) Summary of the experiments design. (**B**) Total disease activity index (DAI) score was measured every day during the experimental period. (**C**–**F**) The plasma levels of NO, ROS, TNF, or IL-1β. Data are expressed as the means ± S.E.M. (*n* = 5–7/group). The statistical significance between values for each group was assessed by Dunnett’s *t*-test. ** *p* < 0.01 between 5% DSS and control groups; ^#^ *p* < 0.05, ^##^ *p* < 0.01 between 5% DSS vs. 60 mg/kg EA groups.

All the animal studies were approved and conducted by the guidelines of the Institutional Animal Care and Use Committee (IACUC) of the Andong National University (approval number: ANU 2020-2-0701-02). Male C57BL/6N mice (8 weeks old, 20–22 g of body weight) were purchased from OrientBio Co. (Seongnam, Republic of Korea). After one week of adaptation, animals were randomly assigned to three experimental groups (*n* = 5–7 mice per group) distributed as follows: (a) control group (CON), who received only drinking water; (b) DSS treatment group (DSS), who received 5% DSS only; and (c) DSS + EA treatment group (DSS + EA), who received 5% DSS and EA (60 mg/kg/day) (Figure 2A). In this mouse model, acute colitis was induced by 5% (*w*/*v*) DSS in the drinking water for 7 days. EA (60 mg/kg/day) was orally administered daily after EA was freshly prepared by suspension in water just before oral administration. The daily EA dose was calculated based on the formula for calculating physiologically and clinically relevant doses [25]. We did not have a group for EA alone, since our observation showed no apparent change caused by EA alone, as recently reported [19,21,22,23,24]. During the experimental period, body weights, stool consistency, and blood in feces of all mice were monitored every day [26]. The blood, liver, cecum, colon, and brain samples were collected for further experiments.

### 2.3. Assessment of Disease Activity Index

Animal body weights, the amounts of consumed water (mL) and food intake (grams), and occurrence of diarrhea were recorded daily throughout the experiments for all indicated mouse groups (Figure 2B). The condition of each colon was scored for microscopically visible damage on a 0–4 scale, according to criteria reported previously by Cooper et al. [27]. The disease activity index (DAI) was determined according to the parameters outlined in Table S1.

### 2.4. Histological Analysis and Plasma AST and ALT Measurement

The mouse tissues (colon, liver, or brain) were fixed immediately in a 10% (*v*/*v*) neutral formalin solution and then paraffin embedded. For histopathological analysis, paraffin blocks were sectioned and stained with hematoxylin and eosin (H&E) dyes. Tissue damages were observed under a polarization microscope (Leica, Bensheim, Germany). The plasma ALT and AST levels in each mouse were determined by using the standard endpoints colorimetric assay kit (Bio Vision, Milpitas, CA, USA), as previously described [28].

### 2.5. Endotoxin, Triglyceride, and Reactive Oxygen Species Measurement

Plasma endotoxin levels were tested by a Pierce™ Chromogenic Endotoxin Quant Kit following the commercial protocol [23,24].

The amounts of hepatic triglyceride (TG) were tested by following the commercial protocol (Asan Co., Ltd., Gimpo, Republic of Korea). The amounts of plasma reactive oxygen species (ROS) were tested by 2′,7′-dichlorofluorescein diacetate (DCFH-DA, Thermo Fisher Scientific, Waltham, MA, USA). The DCFH-DA fluorescence was calculated as previously reported [23,24].

### 2.6. Inflammatory Markers Messurment

The plasma was measured using commercial ELISA kits for interleukin (IL)-1β and tumor necrosis factor (TNF) (R&D systems, Minneapolis, MN, USA) by following the manufacturer’s protocols. NO level was measured using the Nitrate/Nitrite kit Colorimetric Assay Kit (Cayman Chemical Co., Ann Arbor, MI, USA), as previously reported [23,24].

### 2.7. Immunoblot Analysis

Parts of the liver, colon, or brain from each mouse within the same groups were pooled and homogenized with 1x radioimmunoprecipitation assay (RIPA) buffer. The equal amounts of protein were separated on SDS-PAGE and transferred to nitrocellulose membranes (Bio-Rad Laboratories, Inc., Hercules, CA, USA). The nitrocellulose membranes were incubated with primary antibodies (to various target proteins, as indicated in each figure). These blots were incubated with secondary antibodies. Signals were visualized with an enhanced chemiluminescence kit (Thermo Fisher Scientific, Waltham, MA, USA). The intensities of the immuno-reactive bands were quantified by densitometry using Fusion solo (VilBer, Paris, France). The primary antibodies used were as follows: iNOS (1:3000, Cat. No. ab136918, Abcam, Cambridge, UK), 3-nitrotyrosine (1:3000, Cat. No. ab61392, Abcam), cytochrome P450-2E1 (CYP2E1, 1:3000, Cat. No. ab28146, Abcam), COX-2 (1:1000, Cat. No. SC-376861, Santa Cruz, Dallas, TX, USA), p-ERK (1:1000, Cat. No. SC-7383, Santa Cruz), p-JNK (1:1000, Cat. No. SC-6254, Santa Cruz, Dallas, TX, USA), p-P-38 (1:1000, Cat. No. SC-7973, Santa Cruz), p-IκB-α (1:1000, Cat. No. SC-8404, Santa Cruz), p-NF-κB (1:1000, Cat. No. SC-271908, Santa Cruz), ZO-1 (1:3000, Cat. No. ab216880, Abcam), occludin (1:1000, Cat. No. SC-271842, Santa Cruz), E-cadherin (1:1000, Cat. No. SC-8426, Santa Cruz), β-catenin (1:1000, Cat. No. SC-7963, Santa Cruz), γ-catenin (1:1000, Cat. No. SC-59986, Santa Cruz), Bax (1:1000, Cat. No. SC-7480, Santa Cruz), cleaved caspase-3 (1:1000, Cat. No. #9661, Cell Signaling), and GAPDH (1:1000, Cat. No. SC-47724, Santa Cruz).

### 2.8. Apoptosis Assay

Liver specimens were fixed and embedded in paraffin. The terminal deoxynucleotidyl dUTP nick end labeling (TUNEL) analysis was determined by the ApopTag peroxidase in situ apoptosis detection kit (Sigma-Aldrich, Ann Arbor, MI, USA), as previously reported [23,24].

### 2.9. Microbial 16S Sequencing and Bioinformatics

Stool samples were aseptically collected from the cecum of each mouse and rapidly frozen at −80 °C. DNA was extracted using Mag-Bind Universal Pathogen DNA Kit (Chunlab, Seoul, Republic of Korea). DNA sequencing and bioinformatic analyses for bacterial 16S ribosomal RNA of each cecum sample were performed at the Chunlab (https://www.chunlab.com).

### 2.10. Cell Culture and Confocal Microscopy

Mouse neuroblastoma neuro-2a (N2a; CCL-131, American Type Culture Collection, Manassas, VA, USA), T84 human colon cells, and mouse liver AML12 cells were grown in Dulbecco’s modified Eagle’s medium (DMEM) supplemented with 10% heat inactivated fetal bovine serum (FBS) and 1% penicillin–streptomycin.

For detecting ZO-1 or occludin immunofluorescence, T84 human colon cells were stained anti-ZO-1 or occludin antibody and were incubated with Alexa Fluor 488-labeled anti-rabbit secondary antibody (Thermo Fisher Scientific, Waltham, MA, USA).

For detecting CYP2E1 immunofluorescence, AML12 cells were incubated with the anti-CYP2E1 antibody, as indicated, at 4 °C overnight. For immunofluorescence detection, the cells were incubated with Alexa Fluor 488-labeled anti-rabbit secondary antibody (Thermo Fisher Scientific, Waltham, MA, USA).

Cells were incubated with DCFH-DA and cleaved caspase-3. For nuclear staining, the cells were incubated with 1 mg/mL 4′,6′-diamino-2-phenylindole (DAPI) for 5 min. The cells were washed and mounted with VECTASHIELD mounting medium (Vector Laboratories, Burlingame, CA, USA). Fluorescence images were collected by using a microscope.

### 2.11. Statistical Analysis

Data were analyzed using SPSS 26.0 program (SPSS Inc., Chicago, IL, USA), and mean difference of *p* < 0.05 was considered significant. Once significance was recognized, Dunnett’s *t*-test was conducted to compare the difference between groups, as previously reported [23,24].

## 3. Results

### 3.1. EA Attenuated the Inflammatory and Oxidative Stress of DSS-Induced Acute Colitis Mice

To study the anti-inflammatory and antioxidant effects of EA (60 mg/kg/day) in vivo, we used an acute colitis mouse model induced by 5% DSS in drinking water for 7 days (Figure 2A). Symptomatic indexes of colitis, such as body weight loss, mucus, watery diarrhea, and rectal bleeding were evaluated every day during the experimental period (Figure 2B). The disease activity index (DAI) score of the DSS group was considerably increased. However, the DSS + EA group showed a slightly reduced DAI score of these IBD manifestations. To evaluate the changes in inflammatory responses in the DSS-induced mouse colitis, the levels of oxidative stress markers NO and ROS, as well as pro-inflammatory cytokines such as TNF and IL-1β, were determined. The plasma NO and ROS levels of the DSS group were significantly elevated (Figure 2C,D, respectively). However, DSS + EA treatment significantly reduced the levels of plasma NO and ROS. The plasma TNF and IL-1β levels were increased in the DSS group, while their levels were significantly decreased in DSS + EA treatment (Figure 2E,F, respectively). These results suggest that EA administration showed anti-inflammatory and antioxidant activities in DSS-induced colitis mice.

### 3.2. EA Reduced Endotoxin Levels and Changed the Gut Microbiota of DSS-Induced Acute Colitis in Mice

**Figure 3:**
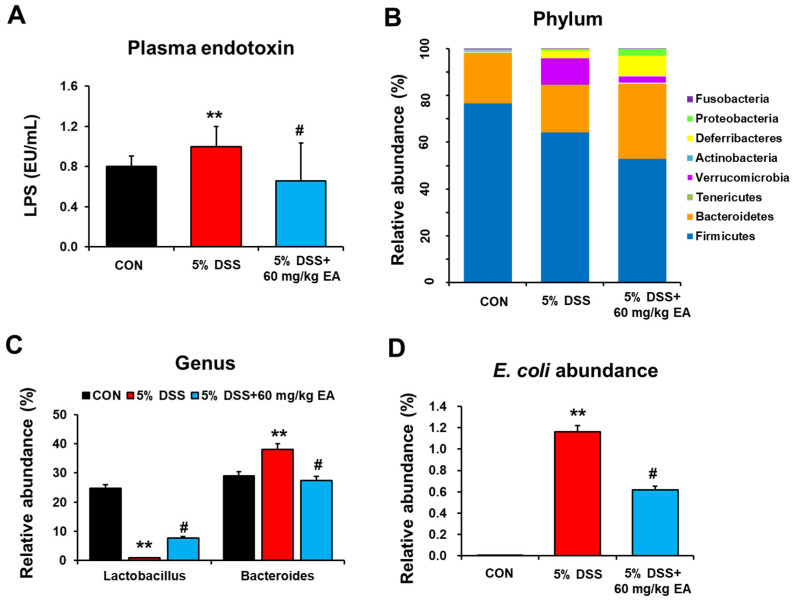
EA prevented DSS-induced gut dysbiosis and leakiness in IBD mice. (**A**) Plasma levels of endotoxin, a plasma marker of gut leakiness. (**B**) Proportional composition and abundance of various bacterial phyla to the overall gut microbiome in the cecum of the indicated groups. (**C**,**D**) The relative abundance of genus *Lactobacillus* and *Bacteroides* and genus *E. coli* are presented for the indicated groups. Data are expressed as the means ± S.E.M. *(n* = 5–7/group). The statistical significance between values for each group was assessed by Dunnett’s *t*-test. ** *p* < 0.001 between 5% DSS and control groups; ^#^ *p* < 0.05 between 5% DSS vs. 60 mg/kg EA groups.

Lipopolysaccharide (LPS) production by Gram-negative bacteria might be increased in DSS-induced colitis and aggravate intestinal tissue damage [29]. DSS exposure caused gut dysbiosis and enhanced leaky-gut-induced bacteremia [29]. Our data revealed that mice with DSS-induced colitis showed significantly elevated endotoxin levels compared to those of control mice, while DSS + EA (60 mg/kg/day) treatment markedly reduced the plasma endotoxin levels (Figure 3A). However, daily treatment with a lower dose of EA (30 mg/kg/day) slightly but not significantly decreased the DSS-mediated elevation of plasma LPS levels (Figure S1). These results suggest a dose-dependent effect of EA against DSS-mediated gut leakiness and endotoxemia. Gut microbiome sequencing analyses showed no difference in phylum composition among the three groups (Figure 3B). In DSS-induced colitis mice, the amounts of *Verrucomicrobia* increased, whereas *Bacteroidetes* abundance decreased at the phylum level (Figure 3B). Interestingly, *Lactobacillus* abundance markedly decreased in DSS treatment and was restored in DSS + EA treatment mice, while opposite trends were observed with *Bacteroides* (Figure 3C). Furthermore, mice with DSS-induced colitis showed an increase in the abundance of *E. coli*, relative to that in control mice, and this elevated *E. coli* was significantly decreased in the DSS + EA treatment mice (Figure 3D). Given that bacterial species within an ecosystem compete for energy resources for survival [30], the observed decrease in gut microbiome diversity in the DSS group might lead to enhanced survival of pathogenic *E. coli*. These results also demonstrate that the EA treatment strategy may work by aiming to reduce potentially harmful gut bacteria and fungi.

### 3.3. EA Reduced Oxidative Stress Marker Proteins and p38 MAPK Phosphorylation in the Colon of DSS-Induced Acute Colitis

**Figure 4:**
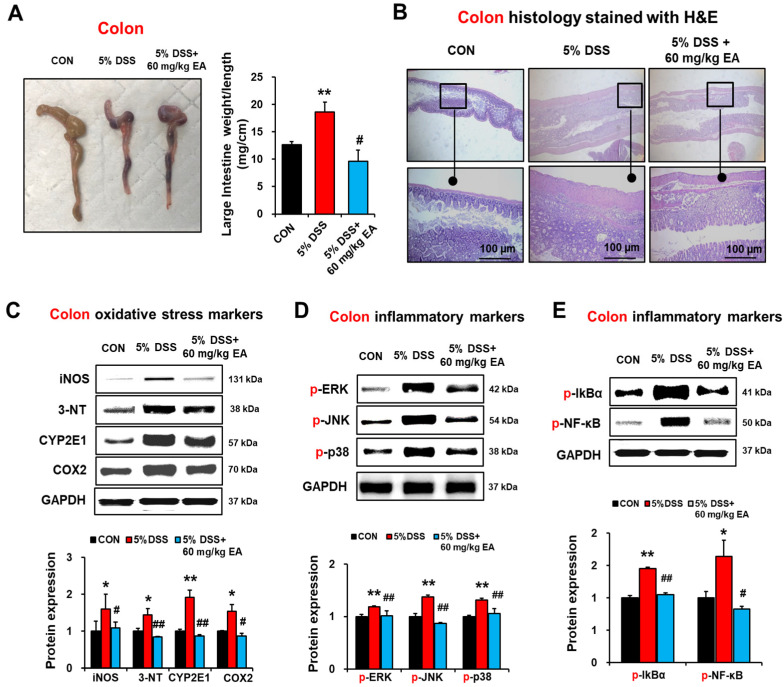
EA treatment reduced oxidative/nitrative stress and NF-κB/MAPK signals in the colon of DSS-induced IBD mice. (**A**) Representative of macroscopic images and length of large intestines, as indicated. (**B**) Representative H/E-stained images of formalin-fixed colon sections in the indicated groups. (**C**) The levels of colon iNOS, nitrated proteins detected by anti-3-NT antibodies, CYP2E1, and COX2 in the indicated groups are presented. (**D**,**E**) The levels of p-ERK, p-JNK, p-p38, p-IκBα, and p-NF-κBp65 in the indicated groups are presented. Data represent means ± S.E.M. (*n* = 5–7/group). The statistical significance between values for each group was assessed by Dunnett’s *t*-test. * *p* < 0.05, ** *p* < 0.01 between 5% DSS and control groups; ^#^ *p* < 0.05, ^##^ *p* < 0.01 between 5% DSS vs. 60 mg/kg EA groups.

The inflammatory changes of the intestinal tract were associated with a significant increase in a weight/length ratio of the mouse colon as an indicator of inflammation. Compared to the CON group, the weight/length ratio of the mouse colon in the DSS group was significantly increased (12.63 mg/cm in the CON group vs. 18.62 mg/cm in the DSS group, *p* < 0.05). However, EA treatment significantly reduced the weight/length ratio of the mouse colon (*p* < 0.05 vs. DSS group) (Figure 4A). Histological examination revealed that marked alterations in membrane architecture, degeneration of intestinal crypts, mucosal atrophy, and infiltration of inflammatory cells were observed in the colons of DSS treatment compared with the CON group (Figure 4B). However, within the DSS + EA group, treatment with EA preserved the extension of crypts and ameliorated inflammatory reactions such as mucosal and submucosal infiltrations. Our current results showed that exposure to 5% DSS significantly elevated the amounts of intestinal iNOS, 3-NT, CYP2E1, and COX-2, whereas EA administration significantly attenuated the elevated levels of these oxidative/nitrative stress marker proteins in DSS-induced colitis mice (Figure 4C).

MAPK signaling pathways play a key role in regulating inflammation [31]. To determine whether EA regulates MAPK signaling in acute DSS-induced colitis, phosphorylation of MAPK isoforms and NF-κB-mediated transcription activation in the colon were evaluated. DSS exposure markedly elevated the levels of p-ERK, p-JNK, p-p38, p-IκBα, and p-NF-κBp65 in colonic tissues (Figure 4D,E). In contrast, EA treatment significantly prevented the elevated levels of p-ERK, p-JNK, p-p38, p-IκBα, and p-NF-κBp65 (Figure 4D,E). These results clearly showed that DSS exposure significantly increased oxidative/nitrative stress marker proteins and NF-κB/MAPK signaling pathways, whereas EA administration significantly blunted the elevated levels of these markers and pro-inflammatory signaling pathways.

### 3.4. EA Restored the Gut Tight Junction and Adherent Junction Proteins in DSS-Induced Acute Colitis Mice

**Figure 5:**
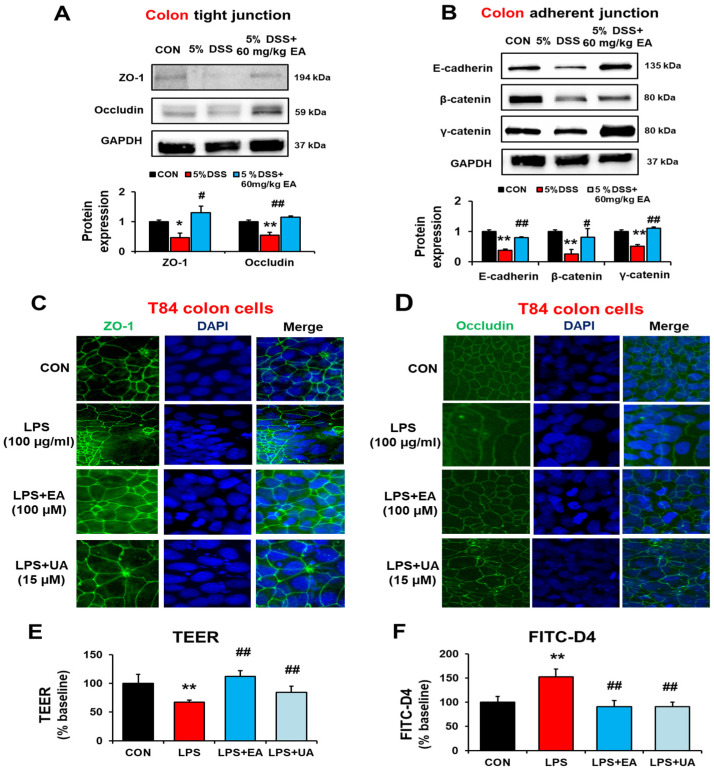
EA treatment prevented DSS-induced intestinal hyper-permeability in IBD mice and T84 colon cells. (**A**,**B**) Representative levels of the designated colon TJ and AJ proteins in the indicated groups are presented. Data represent means ± S.E.M. (*n* = 5–7/group). The statistical significance between values for each group was assessed by Dunnett’s *t*-test. * *p* < 0.05, ** *p* < 0.01 between 5% DSS and control groups; ^#^ *p* < 0.05, ^##^ *p* < 0.01 between 5% DSS vs. 60 mg/kg EA groups. (**C**) T84 cells were treated with culture media (CON) or 100 µg/mL LPS in the absence or presence of 100 µM EA or 15 µM UA for 24 h, as indicated. (**D**) Confocal images showed the disorganized ZO-1 and occludin in LPS-exposed T84 cells, but EA or UA treatment restored ZO-1 and occludin organization. Scale bar is 40 μm. Cell nuclei were counter-stained with DAPI. (**E**,**F**) Representative levels of TEER and permeability to FITC-D4 after 3 h pretreatment without or with the specific agent, EA or UA. The statistical significance between values for each group was assessed by Dunnett’s *t*-test. ** *p* < 0.01 between LPS and control groups; ^##^ *p* < 0.01 between LPS and LPS + EA or LPS + UA groups.

IBD is associated with increased intestinal permeability and decreased expression of gut tight junction (TJ) and adherens junction (AJ) proteins in the inflamed mucosa [32]. Therefore, we studied the effect of EA on the levels of intestinal TJ proteins (e.g., ZO-1, occludin) and AJ proteins (e.g., E-cadherin, β-catenin, γ-catenin) by immunoblot analyses. DSS treatment reduced the levels of TJ and AJ proteins, whereas EA treatment significantly prevented the DSS-mediated decrements of these TJ/AJ proteins (Figure 5A,B, respectively). Taken together, DSS-induced colitis was associated with the decreased levels of colon TJ and AJ proteins with elevated leaky gut and endotoxemia. However, EA treatment significantly blocked the decreased amounts of the intestinal TJ and AJ proteins and gut leakiness in DSS-induced colitis.

It is well established that the endotoxin LPS is a bacteria-derived pro-inflammatory molecule that can damage colon epithelial cell monolayers [33,34]. Based on these reports [33,34] and our previous results [19,21,22,23,24], we also hypothesized that EA could reduce the LPS-induced injury to the T84 colon epithelial cell monolayer. To support our hypothesis, we determined the expression levels of ZO-1 and occludin TJ proteins in human T84 epithelial cells treated with vehicle, LPS, and LPS plus EA by immunohistochemical (IHC) staining. Our IHC results showed that the LPS-induced decreases of ZO-1 and occludin were prevented by treatment with EA or its metabolite, urolithin A (UA) (Figure 5C,D, respectively). In addition, TEER and FITC-dextran measurement data showed that EA or UA treatment prevented LPS-induced gut permeability (Figure 5E,F, respectively). Therefore, we conclude that EA treatment prevented the LPS-induced intestinal barrier dysfunction both in vivo and in vitro models.

### 3.5. EA Prevented Hepatic Injury and Apoptosis in DSS-Induced Acute IBD Mice

**Figure 6:**
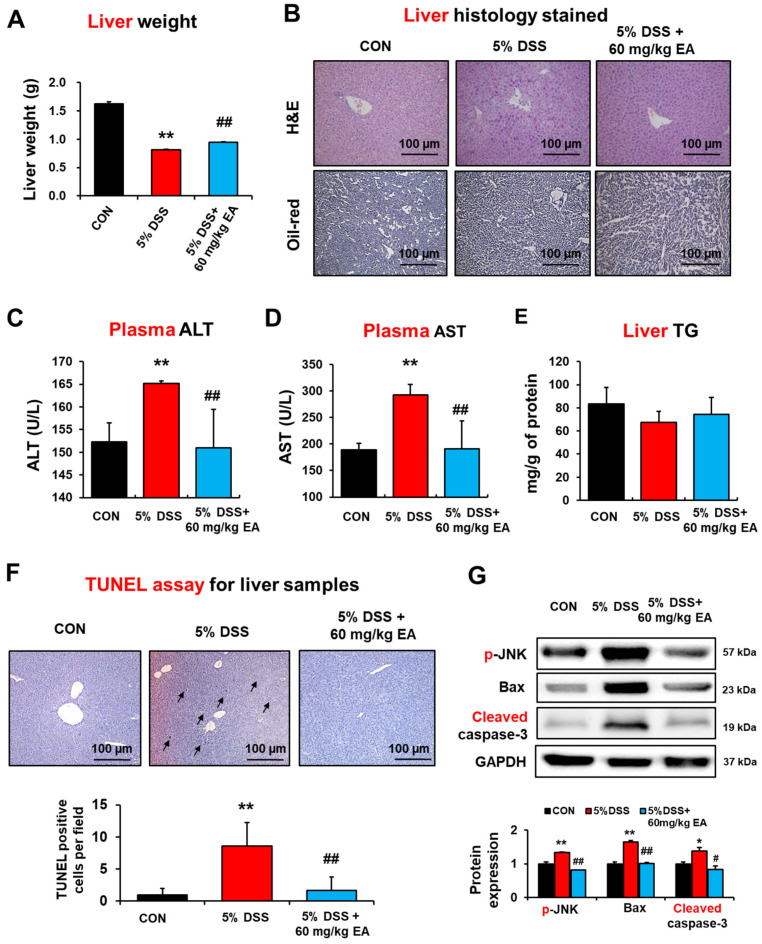
Effects of EA on liver apoptosis markers proteins of liver in DSS-induced IBD mice. (**A**) Liver weight changes. (**B**) Representative H/E-stained formalin-fixed liver sections for CON, DSS, and DSS + EA groups. (**C**–**E**) Plasma ALT, AST, and hepatic triglyceride levels in DSS-induced IBD mice. (**F**) Representative images of TUNEL assay for the indicated groups are presented. Arrows indicate TUNEL positive cells. (**G**) The levels of apoptosis makers in the indicated groups are presented. Data represent means ± S.E.M. *(n* = 5–7). The statistical significance between values for each group was assessed by Dunnett’s *t*-test. * *p* < 0.05, ** *p* < 0.01 between 5% DSS and control groups; ^#^ *p* < 0.05, ^##^ *p* < 0.01 between 5% DSS vs. 60 mg/kg EA groups.

DSS-induced colitis increased portal LPS levels and further enhanced hepatic inflammation and fibrogenesis [35]. Plasma markers of liver injury were determined on day 7 after DSS administration in the absence or presence of EA treatment to further evaluate the preventive effect of EA on DSS-induced liver injury. EA treatment reduced the liver weight and hepatocellular necrosis caused by DSS (Figure 6A,B, respectively). The plasma activities of ALT and AST in the DSS-treated group were significantly elevated compared to the control group, but EA treatment decreased these blood markers of liver injury (Figure 6C,D, respectively). However, oil-red O staining and biochemical measurement revealed no or little changes in the levels of hepatic triglyceride (TG) in DSS-induced liver injury (Figure 6B,E).

Furthermore, histological TUNEL analysis showed markedly elevated apoptosis of hepatocytes and liver injury in the DSS-exposed mice, but this was prevented by EA treatment (Figure 6F). In addition, the levels of apoptosis-related proteins p-JNK, pro-apoptotic Bax, and cleaved (activated) caspase-3 were markedly elevated in the DSS-exposed mice but significantly decreased in the EA-treated group (Figure 6G). Therefore, these results showed that EA treatment significantly prevented the DSS-induced liver damage.

### 3.6. EA Reduced the Expression of Oxidative/Nitrative Stress Marker Proteins and p38 MAPK Phosphorylation in the Liver of DSS-Induced Acute Colitis Mice

**Figure 7:**
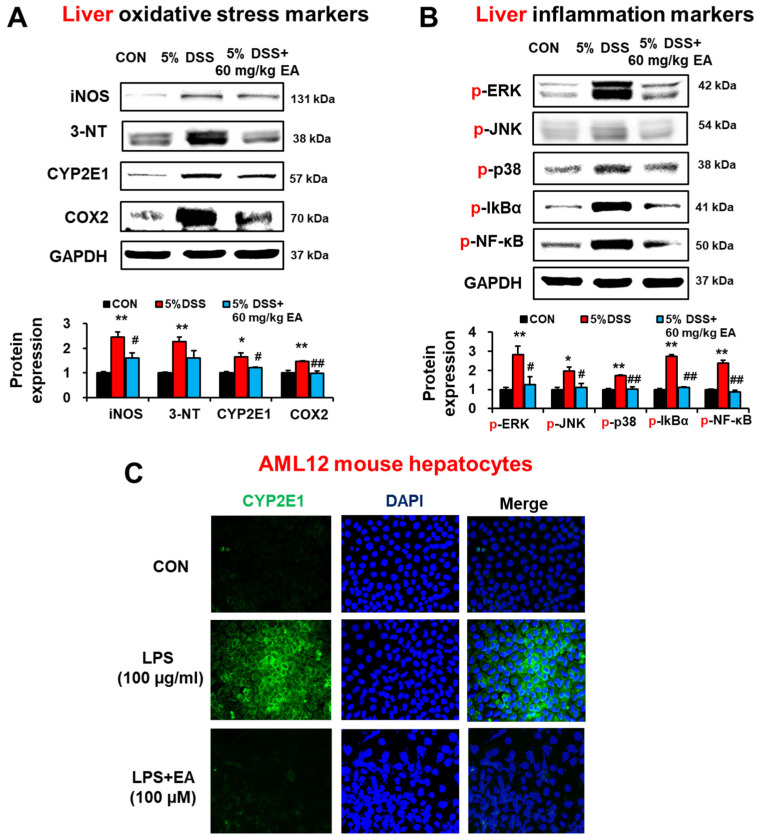
EA treatment reduced the levels of hepatic marker proteins of oxidative stress and inflammation in DSS-induced IBD mice. (**A**,**B**) The levels of oxidative stress markers and inflammatory markers are presented. After 7 days of DSS, livers were harvested, and protein lysates were prepared for immunoblotting analysis. (**C**) Representative images of confocal microscopy CYP2E1 expression in AML12 cells exposed to the LPS in the absence or presence of EA. Scale bar is 10 μm. Data represent means ± S.E.M. (*n* = 5–7/group). The statistical significance between values for each group was assessed by Dunnett’s *t*-test. * *p* < 0.05, ** *p* < 0.01 between 5% DSS and control groups; ^#^ *p* < 0.05, ^##^ *p* < 0.01 between 5% DSS vs. 60 mg/kg EA groups.

The hepatic levels of iNOS, 3-NT, CYP2E1, and COX-2 were elevated in DSS-exposed IBD mice, whereas EA treatment significantly reduced the elevated levels of these oxidative/nitrative stress marker proteins in the DSS-exposed group (Figure 7A). DSS exposure significantly increased phosphorylation (activation) of ERK, JNK, and p-38, but EA treatment significantly inhibited these proteins (Figure 7B).

To determine whether the suppression of inflammation by EA treatment is mediated by regulating the NF-κB pathway, the levels of unphosphorylated or phosphorylated NF-κBp65 and IκB were determined. Immunoblot results showed that phosphorylation of p65 and IκBα was significantly increased in the DSS-exposed group, but prevented by EA co-treatment (Figure 7B). These results indicated that EA treatment prevented elevation of inflammation and oxidative stress through blocking NF-κB activation.

To directly study the preventive role of EA treatment in liver injury, we also studied whether EA could reduce the LPS-mediated CYP2E1 increase in AML12 mouse hepatocytes. Confocal microscopy results showed that the LPS-induced elevation of CYP2E1 was prevented by EA treatment in AML12 mouse hepatocytes (Figure 7C).

### 3.7. EA Reduced the Expression of Oxidative Stress Marker Proteins and Phosphorylation of MAPKs in the Brains of DSS-Induced Acute Colitis Mice

**Figure 8:**
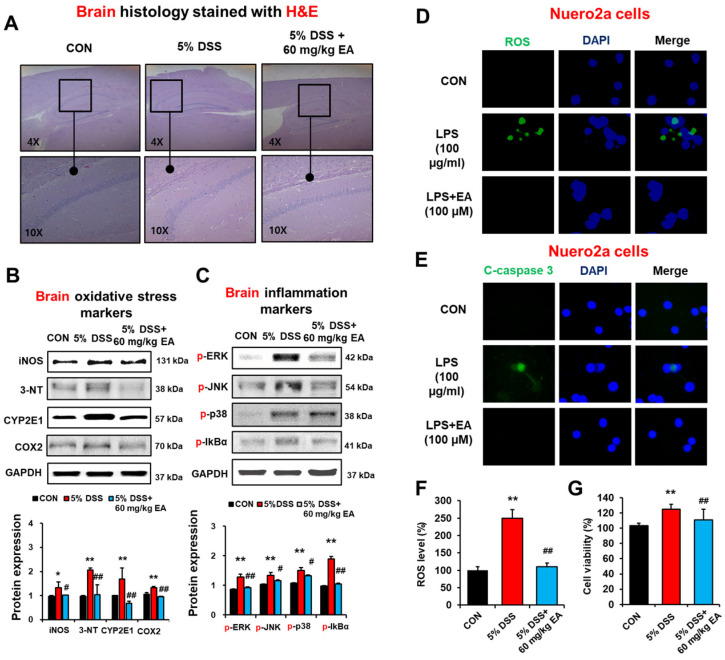
EA treatment reduced brain marker proteins of oxidative stress and inflammation in DSS-induced IBD mice. (**A**) Representative H/E-stained formalin-fixed brain sections for CON, DSS, and DSS + EA groups. (**B**,**C**) The levels of oxidative stress and inflammatory marker proteins are presented. (**D**,**E**) Representative confocal microscopy images of ROS production and cleaved-caspase-3 in neuro-2A cells exposed to the LPS in the absence or presence of EA. Scale bar is 40 μm. (**F**,**G**) The levels of ROS and cell viability in Neuro-2A cells exposed to the LPS in the absence or presence of EA. Data represent means ± S.E.M. (*n* = 5–7/group). The statistical significance between values for each group was assessed by Dunnett’s *t*-test. * *p* < 0.05, ** *p* < 0.01 between 5% DSS and control groups; ^#^ *p* < 0.05, ^##^ *p* < 0.01 between 5% DSS vs. 60 mg/kg EA groups.

To evaluate whether EA treatment can prevent brain injury associated with DSS-induced colitis through the gut–brain axis, we determined the levels of oxidative stress and inflammation marker proteins in the brains of different groups by immunoblotting and histological analyses. Histological evaluation with H&E staining showed no or little difference between DSS and DSS + EA treatment groups (Figure 8A). Our immunoblot results showed that DSS treatment significantly elevated the amounts of iNOS, nitrated proteins detected by 3-NT antibody, COX-2, and CYP2E1 in brain extracts, whereas EA administration significantly blunted the elevated levels of these oxidative/nitrative stress marker proteins (Figure 8B). Furthermore, the levels of phosphorylated (activated) ERK, JNK, and p-38 in brain extracts were significantly increased in DSS-exposed mice, but EA treatment prevented the elevation of these proteins (Figure 8C). In addition, phosphorylation of p65 and IκBα was significantly increased in the DSS-exposed group, but reversed in EA-treated mice (Figure 8C). These results indicated that EA treatment reduced the levels of inflammation and oxidative/nitrative stress marker proteins by regulating the NF-κB activation in the brain.

To directly study the EA-mediated protection in neuronal cell death by LPS, which was elevated via gut leakiness in DSS-exposed mice (Figure 3A), we also studied whether EA can reduce the LPS-induced injury to neuro2A cells. Confocal microscopy results showed that the LPS elevated ROS production and cleaved caspase-3 (c-caspase 3), which was prevented by EA treatment in Neuro2A cells (Figure 8D,E). Similar results of ROS production and cell viability were observed by ROS measurement and MTT analyses (Figure 8F,G). Thus, our data confirmed that EA treatment reduced LPS- or DSS-mediated neuronal cell death.

## 4. Discussion

Inflammatory bowel disease (IBD) represents an idiopathic chronic, relapsing inflammatory disorder in the intestines that is caused by dysregulation of the mucosal immune system [36]. Dietary factors are known to influence the composition and abundance of gut microbiome and have the potential to shape the interplay between gut microbiome and immune responses involved in the pathogenesis of IBD [37,38] and many other diseases. Dietary factors, including bacterial products and gut-derived metabolites, such as short-chain fatty acids, can affect the colonization of certain gut microorganisms [39,40] and gut permeability changes. In the search of potential immunoinflammatory therapeutic strategies, accumulating experimental and epidemiologic reports show beneficial effects of various polyphenols as secondary plant metabolites ubiquitously present in many medicinal plants, fruits, and vegetables on several autoimmune diseases, including IBD [36].

Moreover, polyphenols, despite their very low bioavailability, have indeed shown anti-inflammatory, gut-microbiome-modifying, and antioxidant properties, possibly through preventing gut dysbiosis [41,42], and thus they could aid as supplemental approaches to the already existing therapeutic agents for the management of IBD (i.e., non-steroidal anti-inflammatory drugs).

Retrospective studies have observed a protective effect from fruit and vegetables against IBD [36]. Moreover, polyphenols, despite their very low bioavailability, have indeed shown anti-inflammatory and antioxidant properties possibly through preventing gut dysbiosis [42] and thus could aid as supplemental approaches to the already existing therapeutic agents.

Although we do not know the exact mechanism by which CYP2E1 was elevated in DSS-exposed mice, it is likely that gut dysbiosis with elevated abundance of the ethanol-producing bacteria, including *E. coli*, as shown in Figure 3D, could contribute to the elevated production of ethanol endogenously, leading to the stabilization of CYP2E1 protein in the liver, gut, kidney, and brain [43,44], as recently shown in the case of fructose-exposed rats [22]. However, this needs further confirmation in research.

EA is a natural antioxidant polyphenol compound found in fruits such as berries and pomegranate. The several studies have reported pharmacological properties including antioxidant, anti-inflammatory, and anticarcinogenic activities [45]. A recent report showed that EA can be efficiently delivered to the intestines through a chitosan-conjugated formula [45]. Inflammation and oxidative stress are closely associated with the initiation and progression of many pathophysiological events that are tightly linked with one another [46]. EA has also been shown to exert potent anti-inflammatory activities [47]. For this reason, several studies have emphasized the potential of EA as a candidate for the treatment of many chronic inflammatory diseases and conditions [48,49,50]. Rosillo et al. have revealed for the first time the beneficial effects of EA, a polyphenol presents in some fruits such as pomegranate and raspberries, as well as nuts, in an experimental murine model of Crohn’s disease [45]. In another study, Marín et al. demonstrated that NF-κB was a potential target for the anti-inflammatory effect of EA (0.5% *w*/*w*) supplemented into the normal diet in an ulcerative colitis mouse model [51]. Unlike other studies using an unphysiological level of EA [51], our study clearly demonstrated that orally administered EA (a physiologically relevant dose of 60 mg/kg/day) significantly attenuated the progression of the acute model of colitis in mice, similar to its prevention of binge alcohol-associated liver injury through the gut–liver axis [23,24].

DSS-induced acute colitis model represents one of the most widely used animal models for IBD [52]. The measurement of symptomatic index of colitis, such as body weight loss, mucus, watery diarrhea, and rectal bleeding, is a standard method to evaluate disease progression in the DSS-induced colitis model. In our mouse model of acute colitis, DSS exposure caused marked IBD symptoms, including significant weight loss, the manifestation of diarrhea, and bloody stools, as indicated by the disease activity index (DAI) score (Figure 1). In contrast, EA administration diminished the severity of the intestinal injury induced by DSS. The EA-mediated reduction in the severity of the colitis was accompanied by significant prevention of the loss of weight and the relative weight/length ratio of the colon with good preservation of the extension of crypts and decreased inflammatory reactions such as mucosal and submucosal infiltrations (Figure 4).

In the present study, we evaluated the changes in inflammatory responses in the mouse model of DSS-induced IBD. The macrophages actively participate in inflammatory responses by releasing the pro-inflammatory cytokines IL-1β and IL-6, as well as other inflammatory factors, such as NO in inflammatory responses [53]. In this study, we demonstrated the preventive effects of a physiologically relevant dose of EA on the production and/or secretion of the plasma NO and pro-inflammatory cytokines, such as IL-1β and TNF. Therefore, EA exerts anti-inflammatory and immunomodulatory activity in the DSS-induced IBD model in mice. Although the precise mechanism by which EA decreases serum levels of these pro-inflammatory cytokines is unclear, it is likely that EA acts through direct inhibition of the NF-κB pathway, as shown in this study and reported in the renal disease model [54]. According to a recent report [55], EA can form a stable complex with polysaccharides. In this regard, we also need to confirm whether EA forms a complex with DSS, leading to its decreased ability to induce colitis.

NF-κB is a key redox-sensitive pro-inflammatory transcription factor. The IκBα is phosphorylated and degraded, while the NF-κB is released and translocated to the nucleus to induce transcriptional expression of many pro-inflammatory cytokines, chemokines, and proteins, including iNOS and COX-2. In this study, suppression of phosphorylation of IκBα by EA treatment led to NF-κB inactivation in a DSS-induced IBD mouse model. Overall, these results indicate that EA administration exhibited antioxidative and anti-inflammatory activities against tissue injury in the gut, liver, and brain of DSS-induced IBD mice. We believe that the beneficial effects of EA can be attributed to its ability to prevent IκBα degradation. Similar results for EA as an anti-inflammatory agent through modulation of the NF-κB activation pathway have been observed in several other studies [56,57,58,59,60].

It is well established that LPS causes inflammation and oxidative stress in many tissues, including the liver and brain. Gut leakiness and gut dysbiosis can induce IBD by increasing the concentrations of LPS and other harmful bacterial products/metabolites. In addition, it is known that various pathological conditions, including neurodegenerative diseases, can be stimulated through the gut–liver–brain axis in patients with IBD. In this study, our results showed that LPS, which can be leaked from the gut through intestinal barrier dysfunction, can increase oxidative stress and inflammation in the liver and brain through the gut–liver–brain axis. The LPS interacts and activates the TLR-4/HMGB-1 signaling pathway, increasing production of pro-inflammatory cytokines (e.g., TNF, IL-1β, and IL-6) and oxidative stress maker proteins (iNOS and COX-2) in hepatic cell lines and mouse liver [61,62]. Furthermore, elevated LPS can impair and passes through the blood–brain barrier (BBB), causing chronic inflammation and neuronal death through increasing the expression of pro-inflammatory cytokines (e.g., TNF, COX-2, and IL-1β) [63,64].

In conclusion, our current study shows that EA, at a physiologically relevant dose, significantly improves systemic symptoms along the gut–liver–brain axis (Figure 1) in DSS-induced IBD mice by suppressing the expression of pro-inflammatory cytokines and inflammatory mediators through preventing the activation of NF-κB and MAPK pathways, oxidative/nitrative stress, gut dysbiosis, and intestinal barrier dysfunction. Based on our results, we therefore suggest that EA deserves further consideration as a potential alternative therapeutic agent for the treatment of inflammatory diseases such as colitis.

## Data Availability

The data presented in this study are available.

## Supplementary Materials

The following supporting information can be downloaded at https://www.mdpi.com/article/10.3390/antiox12101886/s1. Table S1. Scoring system to calculate the disease activity index (DAI). Figure S1. Daily treatment with a lower dose of EA (30 mg/kg/day) slightly but not significantly decreased the plasma LPS level markedly elevated in DSS-treated mice.
