# Supplementary material for: Ellagic Acid Prevented Dextran-Sodium-Sulfate-Induced Colitis, Liver, and Brain Injury through Gut Microbiome Changes
Source: Antioxidants (Basel). 2023 Oct 20;12(10):1886. doi: 10.3390/antiox12101886 (PMC10604018; doi:10.3390/antiox12101886)
Supplement: Supplementary file 1 [file antioxidants-12-01886-s001.zip › antioxidants-2599387-supplementary.pdf]

Supplementary Figure

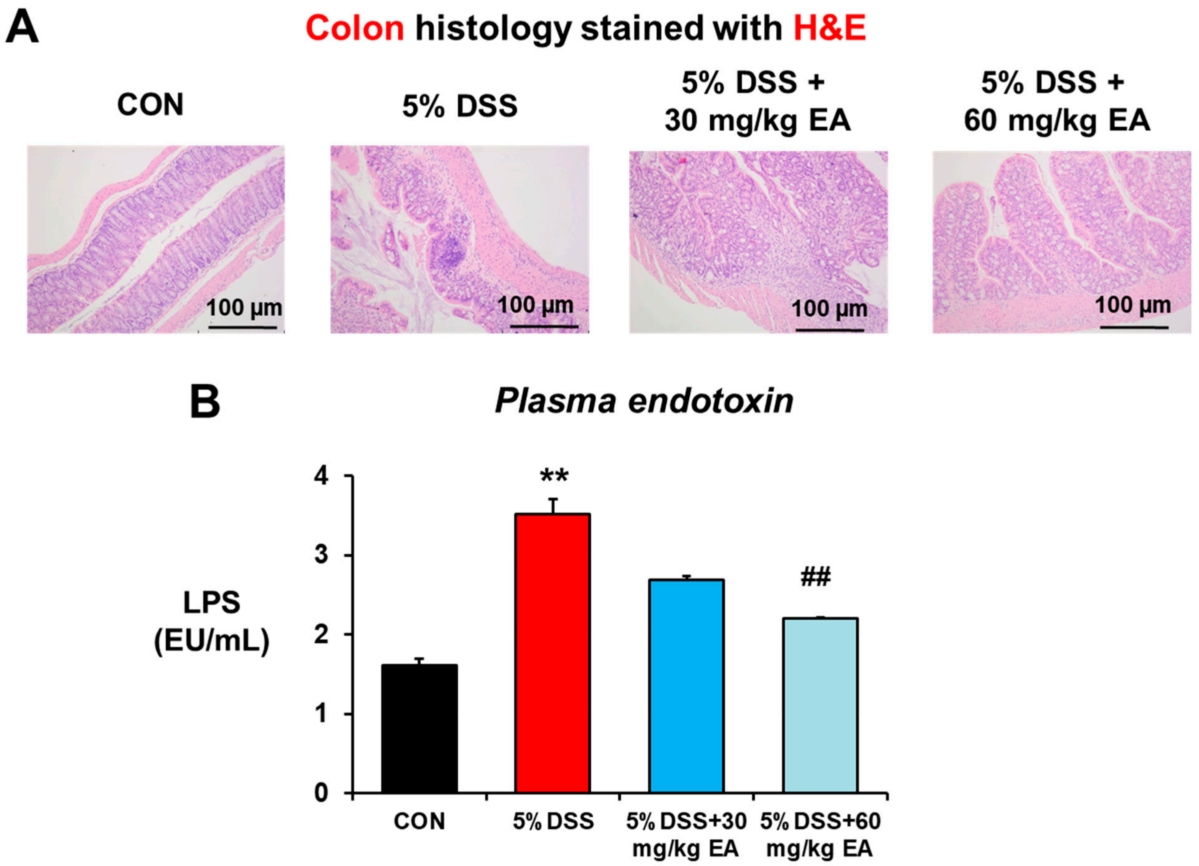

Figure S1. Daily treatment with a lower dose of EA (30 mg/kg/day) slightly but not significantly decreased the plasma LPS level markedly elevated in DSS-treated mice. (A) Representative H/E-stained images of formalin-fixed colon sections in the indicated groups. (B) Plasma levels of endotoxin, as a marker of gut leakiness. Data are expressed as the means ± S.E.M. (n=5-7/group). The statistical significance between values for each group was assessed by Dunnett's t-test. $^{**}P<0.001$ between 5% DSS and control groups; $^{\#\#}P<0.05$ between 5% DSS vs. 60 mg/kg EA groups.

Supplementary Table S1

Supplementary Table S1. Scoring system to calculate the disease activity index (DAI). The DAI value is calculated as the sum of scores of weight loss, steel consistency, and blood feces.

Score	Weight loss	Stool consistency	Visible blood feces
0	None	Normal	None
1	1~5%		
2	6~10%	Loose	Slight bleeding
3	11~20%		
4	<20%	Diarrhea	Gross bleeding
